# Single-cell RNA sequencing of adult rat testes after Leydig cell elimination and restoration

**DOI:** 10.1038/s41597-022-01225-5

**Published:** 2022-03-25

**Authors:** Xiaoju Guan, Minpeng Ji, Xin Wen, Fu Huang, Xingyi Zhao, Dan Chen, Jingjing Shao, Jiexia Wang, Jiajia Xie, Jing Tian, Han Lin, Ping Duan, Barry R. Zirkin, Zhijian Su, Haolin Chen

**Affiliations:** 1 Zhejiang Provincial Key Laboratory of Anesthesiology, Department of Anesthesiology, the Second Affiliated Hospital and Yuying Children’s Hospital of Wenzhou Medical University, Wenzhou, Zhejiang 325027 China; 2 Department of Gynecology and Obstetrics, the Second Affiliated Hospital and Yuying Children’s Hospital of Wenzhou Medical University, Wenzhou, Zhejiang 325027 China; 3 Department of Pharmacology, the Second Affiliated Hospital and Yuying Children’s Hospital of Wenzhou Medical University, Wenzhou, Zhejiang 325027 China; 4 Department of Biochemistry and Molecular Biology, Johns Hopkins Bloomberg School of Public Health, Baltimore, MD 21205 United States of America; 5 Guangdong Provincial Key Laboratory of Bioengineering Medicine, Department of Cell Biology, Jinan University, Guangzhou, China

**Keywords:** Spermatogenesis, Meiosis, Infertility

## Abstract

Spermatogenesis is an efficient, complex, and highly organized proliferation and differentiation process that relies on multiple factors including testosterone produced by the Leydig cells. Although the critical role played by testosterone in spermatogenesis is well recognized, the mechanism by which it works is still not completely understood, partially due to the inability to specifically and precisely monitor testosterone-dependent changes within developing germ cells. Here we present single-cell RNA sequencing data from10,983 adult rat testicular cells after the rats were treated with ethanedimethanesulfonate, which temporarily eliminates Leydig cells. The elimination and recovery of Leydig cells represented a complete testosterone depletion and restoration cycle. The dataset, which includes all developing germ cells from spermatogonia to spermatozoa, should prove useful for characterizing developing germ cells, their regulatory networks, and novel cell-specific markers. The dataset should be particularly useful for exploring the effects of the androgen environment on the regulation of spermatogenesis. As this is the first single-cell RNA-Seq dataset for rat testes, it can also serve as a reference for future studies.

## Abstract (summary)

**Table:** 

Measurement(s)	RNA sequence of rat testicular cells
Technology Type(s)	scRNA-seq
Factor Type(s)	Time course in weeks
Sample Characteristic - Organism	Sprague Dawley Rattus

## Background & Summary

Spermatogenesis is a continuous cell proliferation and differentiation process that yields mature male reproductive cells, i.e. spermatozoa. The process is regulated by both circulatory and local factors. Within the testicular seminiferous tubules, Sertoli cells are the only somatic cells in direct contact with germ cells, providing a supporting microenvironment for germ cell development^1,2^. In addition to Sertoli cells, the Leydig cells play a critical role in regulating spermatogenesis by providing paracrine factors, most importantly testosterone^3^. It is well-established that testosterone is essential for the quantitative maintenance of spermatogenesis^4^. However, the exact mechanism by which testosterone regulates the critical developmental steps of spermatogenesis, whether by targeting specific stages or through affecting specific groups of genes in particular cells, is still largely unknown^5^.

Since germ cells do not express androgen receptors themselves, it is believed that the effects of androgens on spermatogenesis are mediated by nearby somatic cell populations, the Sertoli, Leydig and myoid cells that do have androgen receptors^5^.Most previous studies on androgen effects focused largely on one or more of these somatic cell populations. Little is known about the exact consequences of androgen deprivation on germ cells, including whether there are developmental stages of germ cells that are particularly susceptible to androgen withdrawal, and what the transcriptomic consequences are to germ cells in response to insufficient androgen. Questions such as these are difficult to address at the single-cell level using classical cell biology tools. However, in the last few years, single-cell RNA sequencing (scRNA-seq) has been used successfully to classify and characterize complex cell populations in the mammalian testis^6–12^.

The rat has served as an important model animal for understanding the regulation of spermatogenesis. As in the human, male rats express androgen-binding protein (ABP), a protein that is homologous to sex hormone-binding protein (SHBG) in the human. These proteins play important roles in regulating the transport, tissue delivery, bioactivity, and metabolism of androgens in males. Mice do not produce ABP at meaningful levels (less than 5% of rat production)^13^. By lacking such binding capacity, spermatogenesis can be maintained in mice at extremely low testosterone levels^14,15^, while much higher intratesticular testosterone levels are required for maintaining spermatogenesis in human and rat^4,16,17^.

With the intent to increase understanding of the cellular basis of the hormonal regulation of spermatogenesis, we performed single-cell transcriptomic sequencing of 10,983 adult rat testicular cells that were derived from animals from four different androgen environments: (1) control rats with normal physiological testosterone levels; (2) rats in which Leydig cells were eliminated by ethane dimethanesulfonate (EDS) one week prior to sequencing, and therefore with no detectable serum testosterone; (3) rats 3 weeks post-EDS treatment that contained about 25% of normal testosterone levels due to partial Leydig cell regeneration; and (4) rats 7 weeks post-EDS treatment that had full restoration of Leydig cell populations and normal testosterone levels. The data captured the full developmental stages of spermatogenesis, including meiosis and spermiogenesis, with high data quality (median gene numbers per cell of about 5,000 with very little bench-effect). These data, which can be used for germ cell characterization, marker discovery, stage- and development-dependent gene identification, and network inference analysis, will allow unbiased and novel insights into the molecular and cellular details of spermatogenesis. Given the experimental design, the results should be particularly useful in illuminating the effects of the androgen environment on spermatogenesis.

## Methods

### Animals and treatments

Adult male Sprague Dawley rats 90 days of age were purchased from Shanghai Animal Centre (Shanghai, China). Rats were housed in the animal facilities of the Second Affiliated Hospital of Wenzhou Medical University at 22 °C, 12-hour light, 12-hour dark cycle with free access to water and rat chow. All animal procedures were approved by the Institutional Animal Care and Use Committee of Wenzhou Medical University and were performed in accordance with the Guide for the Care and Use of Laboratory Animals of NIH (NIH publication #85-23, revised in 1985).

To eliminate Leydig cells from the testes, rats (N = 9) were injected with a single dose of ethanedimethanesulfonate (EDS; i.p., 80 mg/kg of BW) dissolved in a mixture of DMSO:PBS (1:3) as previously described^18^. Three rats were treated with DMSO:PBS vehicle as controls. Testes from 3 animals were collected at each of 1, 3, and 7 weeks post-EDS treatment (E1W, E3W and E7W, respectively), or at 7 weeks post vehicle treatment (control, C). This treatment protocol was selected because there is complete Leydig cell elimination by 1 week post-EDS, partial Leydig cell regeneration by 3 weeks post-EDS, and complete regeneration at 7 weeks^18^. The treatments were arranged in such a way that all animals were ready for tissue collection on the same day. Serum testosterone concentrations were assayed using the Immulite 2000 Total Testosterone Assay Kit (Siemens, Germany) with a detection sensitivity of 0.15 ng/ml and intra-assay coefficient of variation of 8.3%.

### Preparation of testicular cell suspensions

To eliminate possible contamination from blood cells, the testicular artery was cannulated and perfused with culture medium M199 containing 2.2 g/l Hepes, 0.1% BSA, and 0.7 g/l sodium bicarbonate (pH 7.4) to clean out blood^19^. After decapsulation, the testes from 3 animals were pooled and then digested with 1 mg/ml collagenase-IV in DMEM/F12 medium at 34°C for 30 min with slow shaking (90 cycles/min), as described previously^19^. After allowing the undigested tissue to settle for 30 seconds, the dispersed cells in supernatants were filtrated through 2 layers (100 µm pore on top of 30 µm pore) of nylon mesh and washed twice with PBS. Cell viability, assayed by 0.4% trypan blue exclusion, was consistently above 85% for all 4 groups.

### Single-cell transcriptomes sequenced by 10X Genomics Chromium

Cell capture, 10x Genomics library preparation, and sequencing were done by Novogene (Beijing, China). After washing twice in PBS, ~7,000 cells were loaded onto 10x Chromium chips with 3′v2 chemistry and barcode to achieve a targeted cell count of 4,000, according to the manufacturer’s instructions (10x Genomics, Pleasanton, CA). After cDNA synthesis, 14 amplification cycles were carried out for each library preparation. The resultant libraries were sequenced using 2 × 150 paired-end sequencing protocol on an Illumina NovaSeq. 6000 platform (Illumina, San Diego, CA), with a read length of 26 bp for cell barcode and unique molecule identifier (UMI) (read 1), 8 bp i7 index read (sample barcode), and 98 bp for actual RNA read (read 2). Each treatment group yielded approximately 550 M reads. All downstream single-cell analyses were performed using Cell Ranger and Seurat software.

### Alignment, barcode assignment and UMI counting

For the purpose of quality control, we used FastQC to perform basic statistics on the quality of the raw reads. Demultiplexed raw sequencing reads were processed and aligned to the rat genome NCBI Rnor6.0 by the 10x Genomics Cell Ranger (v2.1.1) pipeline to generate the filtered gene-barcode matrix containing valid cell barcodes and transcript UMI counts. Only the reads that were confidently mapped to the transcriptome were used for UMI counting. For each gene and each cell barcode, UMIs were counted to construct digital expression matrices, which were filtered a second time using Seurat software with the following two criteria: a gene with expression in more than 3 cells was considered as expressed, and each cell was required to express at least 200 such genes to be counted. Datasets from different treatment groups were integrated using the Cellranger aggr command based on mapped read counts to normalize sequencing depth, producing a single feature-barcode matrix containing all data and clustering models.

### PCA and t-SNE analysis

In order to reduce the gene expression matrix to its most important features, Cell Ranger uses Principal Components Analysis (PCA) to change the dimensionality of the dataset from cells x genes to cells x M where M is a user-selectable number of principal components. For visualizing data in 2D space, Cell Ranger passes the PCA-reduced data into t-Stochastic Neighbor Embedding (t-SNE). Cell Ranger uses two different methods for clustering cells by expression similarity, both of which operate in the PCA space: 1) k-means clustering that groups cells into a pre-set number of clusters; and 2) graph-based clustering that builds a nearest-neighbor graph, where cells are linked if they are among the k nearest Euclidean neighbors. It is then followed by Louvain Modularity Optimization, an algorithm which seeks to find highly connected “modules” in the graph^20^. The Loupe Cell Browser v5.0.0 (10x Genomics) was used to visualize t-SNE projections and “Violin-distributions”.

## Data Records

The raw data in fastq (.fq) format have been deposited on the repository GSA that can be accessed through the project number CRA004852^21^. Expression matrices (*.mtx) and differentially expressed gene (DEG) lists are deposited on the repository OMIX with project number PRJCA006139^22^. A copy of the mtx data is also deposited at figshare^23^. These matrices contain columns and rows corresponding to cells and genes, respectively. The identifiers for the columns and rows are included as separate files (barcodes.tsv and genes.tsv). These processed files correspond to the output produced by the cellranger pipeline.

## Technical Validation

**Fig. 1:**
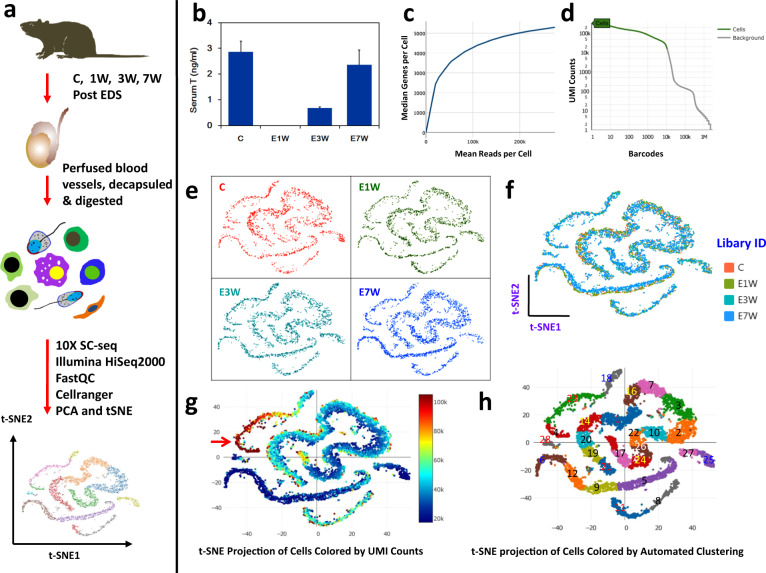
scRNA-seq analysis of rat testicular cells with and without EDS treatment. (**a**) Schematic illustration of the experimental workflow. (**b**) Serum testosterone levels from control (**c**), 1 week (E1W), 3 weeks (E3W), and 7 weeks (E7W) post-EDS treated rats. (c) relationship between Mean Reads per Cell and Median Genes per Cell of C group. (**d**) Relationship between Barcodes and UMI counts of the C group. (**e**) t-SNE projections of testicular cells from the 4 treatment groups. (**f**) Overlayed t-SNE projections of the 4 treatment groups. (**g**) t-SNE projection of cells colored by UMI counts. (**h**) Graph-based clustering of all testicular cells. t-SNE, t-distributed stochastic neighbor embedding.

We employed the 10x Genomics Chromium platform to construct single-cell RNA-seq libraries that were sequenced on an Illumina HiSeq PE150 platform (Fig. 1a). The experiment contained four testis treatment groups that covered a complete Leydig cell depletion and replacement cycle in the adult rat. The process of EDS-induced Leydig cell elimination (E1W) and regeneration (E3W and E7W) was validated by serum testosterone assay (Fig. 1b). For the sequencing process, saturation curve analysis indicated that the sequencing depths were comparable among the 4 treatment groups and were sufficient to detect the highest number of genes per cell (Fig. 1c,d, S1). Detailed data quality metrics for sequencing are listed in Table 1. The total number of reads was above 540 M for all treatment groups. The valid barcodes detected were between 96.7% to 97.0%. The reads mapped confidently to the genome were all between 87.3% to 97.3%, while reads mapped confidently to the transcriptome were between 57.1% to 57.5%. The metrics were very similar among the 4 treatments, reflecting little bias introduced for technical reasons.

**Table 1:** Detailed QC of FASTQ files.

Sample ID	Number of Reads (K)	Valid Barcodes (%)	Q20 Bases in RNA Read (%)	Q30 Bases in RNA Read (%)	GC Content (%)	Reads Mapped Confidently to Genome (%)	Reads Mapped Confidently to Exonic Regions (%)	Reads Mapped Confidently to Transcriptome(%)
C	592,752	97.0	95.5	89.8	46.9	87.3	63.8	57.3
E1W	540,734	96.7	95.8	90.3	47.0	97.3	63.8	57.1
E3W	569,130	96.8	95.4	89.5	47.2	87.4	64.3	57.5
E7W	566,156	96.7	95.4	89.6	47.4	87.8	64.7	57.5

The detailed sequencing statistics based on cells are listed in Table 2 and Figs. 1d and S2–S5. The estimated numbers of cells acquired were between 2,157 to 3,693. Median UMI counts per cell were between 36,562 to 55,914, while median genes per cell detected were between 4,586 and 5,297. These metrics were very similar among the 4 treatment groups except E7W, which captured more cells than the others. The percentage of mitochondrial transcripts is a rough indicator of apoptotic cells, while the percentage of HB measures contaminating red blood cells. In our experiment, very few cells surpassed the commonly used threshold of 5% mitochondrial transcripts and 0.01% HB transcripts, indicating high quality of pure and living cells (Figure S6). Overall, except for slightly lower median genes per cell detected by E7W due to more cells in the group, other metrics were very similar among the 4 groups, reflecting little technical variation.

**Table 2:** Sequencing statistics based on cells.

Sample ID	Estimated Number of Cells	Fraction Reads in Cells	Mean Reads per Cell	Median Genes per Cell	Total Genes Detected	Median UMI Counts per Cell	Sequencing Saturation
C	2,157	84.7%	274,804	5,297	19,131	55,914	50.3%
E1W	2,294	84.6%	235,716	5,183	19,153	52,393	45.0%
E3W	2,839	84.1%	200,468	4,925	19,284	44,653	42.9%
E7W	3,693	84.1%	153.305	4,586	19,324	36,562	39.2%

**Fig. 2:**
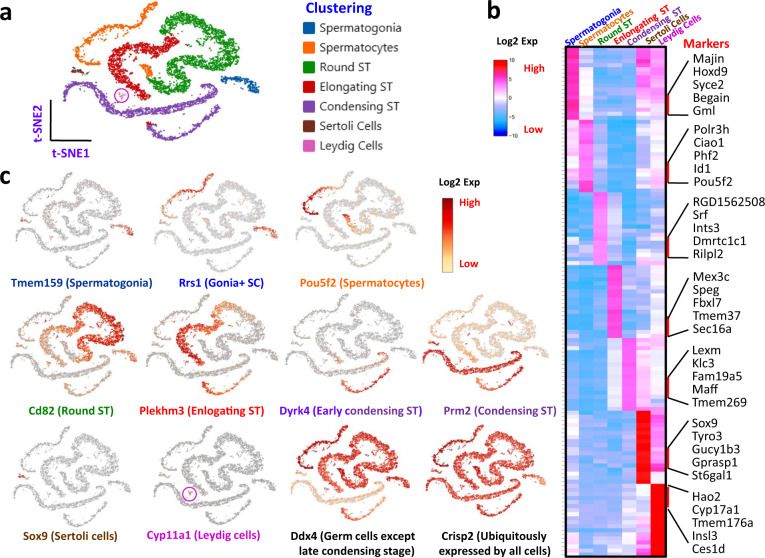
Overview of markers and attributes of the testicular cell types identified by scRNA-seq. (**a**) t-SNE projection of 7 cell types of rat testes. (**b**) heatmap shows the top markers associated with the 7 major cell types identified. (**c**) marker gene expressions (Log2 expression levels) showed by t-SNE projections. Labels for the 7 cell types were color-coded in panel a, and the colors were consistently used for labeling here and in Fig. 3a.

To compare data consistency among the 4 treatment groups further, the feature dimensions of the groups were reduced by PCA and then projected by t-SNE in 2 dimensions (Fig. 1f). Surprisingly, no significant difference was observed between the cell distributions among groups (Fig. 1e). However, enrichment of both the gene count and UMI count was expected in this group^24^, as various germ cell populations in the testis have highly variable RNA content, with the highest levels found in pachytene spermatocytes. This was reflected by the data (Figs. 1g and 2). The section that contained the richest detectable genes (Fig. 1g, red arrow) was exactly the area where pachytene spermatocytes are located (Fig. 2a,c). With automated graph clustering, 28 subclusters were revealed, suggesting a rich potential for the dataset in the identification and characterization of developing germ cell stages, novel markers associated with the stages, and the specific paracrine factors involved among the cells (Fig. 1h).

**Fig. 3:**
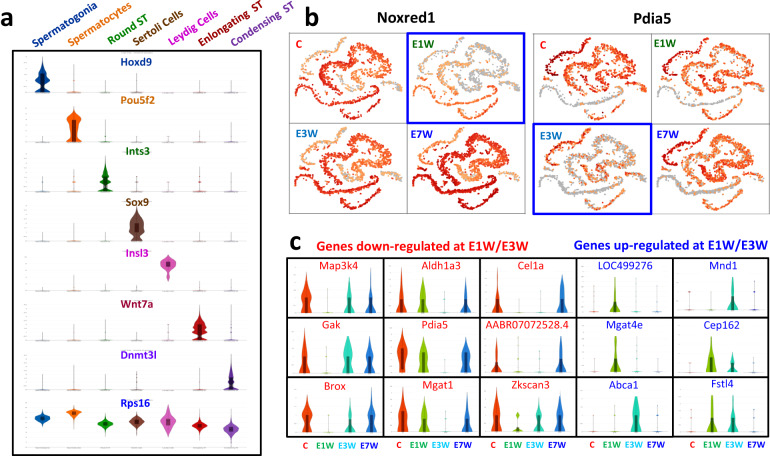
Distributions of marker genes and testosterone sensitive genes. (**a**) Violin-distributions of identified marker genes exclusively expressed by each cell type. Rps16 was ubiquitously expressed by all the cell types. (**b**) Group-dependent t-SNE projections showed expression of testosterone-sensitive genes. Noxred1 and Pdia5 were down-regulated in testosterone-depleted (E1W) or -reduced (E3W) animals (blue boxes). (**c**) Violin-distributions of representative Sertoli cell genes whose expressions were either down- or up-regulated at E1W and/or E3W.

As a way to show the quality of the data, we did a preliminary clustering of cells based on both the graph-mode and a pre-defined number of clusters. With the help of available testicular cell markers from rat^25^, mouse^6–10^ and human^9–12^, we were able to combine the 28 graph-based clusters into 7 major cell types containing 5 germ cell stages, corresponding to spermatogonia (SPG), spermatocytes (SPC), round spermatids (RSPT), elongating spermatids (ESPT), condensed spermatids (CSPT), and 2 somatic cell types, Sertoli and Leydig (Fig. 2a). The top 18 differentially expressed genes for each cell type are summarized by heatmap (Fig. 2b), among which 5 representative genes are displayed by name. A few specific markers for the cluster are also displayed with either t-SNE Projection (Fig. 2c) or Violin-Distribution (Fig. 3a). While most markers only labelled a specific developing germ cell stage or a specific somatic cell type, other genes, such as the well-known germ cell marker Ddx4, marked the whole germ cell population except the late condensing stage. Also, other genes, like Crisp2 or Rps16, were expressed ubiquitously by all testicular cell types.

To boost confidence in the cell classifications, we analyzed the expression patterns of well-known marker genes for each cell type (Supplemental Figures S7-S8). Dazl^7,10,26^, Sohlh2^27,28^ and Elavl3^10^ are well-known SPG markers that were exclusively expressed by the cluster in the current study (Figure S7). Similarly, Phf2^7,28^, Id1^28^ and Ngfr^10^ were found enriched in SPC, while Lrat and Spag6 were enriched in SPT^10^. Tnp1, Tnp2, Prm1 and Prm2 are all well-known markers for elongating to condensed SPT^7,10^. All these were found specifically expressed by the corresponding clusters in the current study (Figures S7, S8). Also, the 2 major somatic populations, Sertoli and Leydig cells, exclusively expressed well-known markers Clu and Gstm6^29^ for Sertoli cells and Cyp17a1 and Cyp11a1 for Leydig cells. All these specific expression markers support the conclusion that the classifications of the cells are accurate.

However, several well-known SPG stem cell markers, including Stra8 and Zbtb16, were not expressed by any clusters. A reason could be that there were too few SPG stem cells included in the samples which prevented them from participating in the SPG cluster. This under-representation of early germ cells is supported by comparison of the ratios of SPG, SPC and SPT of the current study with previously published numbers *in vivo*^30,31^. Compared to numbers of SPTs, both SPG and SPC were represented at only about 40% and 30% levels, respectively. This result represents a limitation of this analysis.

Another well-known SPG stem cell gene with unexpected expression pattern is Gfra1. Instead of being expressed by SPG, as reported by a number of previous studies^7,10,25^, the gene was highly expressed by SPT (Figure S9A, B) instead. This unexpectedly high expression by SPT in rat is supported by a previous microarray study^25^ (Figure S9C). Whether this unexpected expression by SPT in addition to SPG is a rat species-specific phenomenon is still unclear since it is observed, but not discussed, in other species too, including mouse^7,10^ and man^10^.

To further examine the extent to which our classification of rat testis cells is consistent with recent studies of mouse and human testis cells^7,10^, we calculated the number of shared genes for each cell type among the top 200 enriched genes of our study and the lists of the genes of published studies^7,10^. As shown in Supplemental Table S1, there were matches in the number of DEGs for each cell type between our study and that of Green *et al*.^7^. More genes were matched for somatic cells than for germ cells, while the late developing germ cells matched more genes than the early stages. There were far fewer genes shared among different cell types than between the same cell types. This is also true for all the cells between our and Hermann’s mouse and human studies^10^, except mouse SPC (Supplemental Tables S1 and S2). These results indicate that except for mouse SPC, there were high matches found for every other cell type among the 3 studies, suggesting common developmental and regulatory mechanisms in spermatogenesis across the different species. Additional cell type-specific genes can be found in Supplemental Figures S7-S8.

A significant feature of this study’s experimental design is the depletion and restoration of testosterone within the testis. Such a dynamic cycle provides the opportunity to dissect the effects of testosterone on the maintenance and restoration of spermatogenesis. To examine whether depletion and restoration of testosterone impacted any gene’s expression in germ cells, gene expression was compared among the 4 treatment groups (Fig. 3b). We found that genes such as Noxred1 and Pdia5 were inhibited (blue boxes) by testosterone withdrawal at 1 (E1W) or 3 (E3W) weeks but returned to normal levels by 7 weeks (E7W). Interestingly, Noxred1 rebounded by week 1, while Pdia5 rebounded by week 3, suggesting that different regulatory mechanisms may be involved. Moreover, changes in both genes were predominantly within the round spermatid population, though the expression of these two genes was not confined to this population. In addition to germ cells, there were a number of genes expressed differentially by Sertoli cells in response to EDS treatment (Fig. 3c). There were genes that were completely inhibited at 1 and/or 3 weeks after EDS treatment that were completely recovered to control (C) levels by week 7. Also, there were genes not expressed by control animals that were turned on at 1 and/or 3 weeks after EDS treatment. These genes were turned off by week 7 after EDS treatment. Overall, these primary analyses of the dataset further support the quality of the data and the potential for exploring new germ cell features, markers associated with specific developmental stages, and paracrine factors in cell-cell interactions, especially for the role played by testosterone in spermatogenesis.

## Usage Notes

This dataset could be utilized, among other possibilities, to effectively (1) identify and validate new genes and transcripts in rat testicular cells at the single-cell level, (2) develop a more comprehensive annotation system for single-cell transcriptomes of rat testis, (3) identify novel gene regulatory networks related to specific spermatogenic stages in rat, and (4) discover testosterone-dependent genes in developing germ cells and somatic cells.

It should be noted that there are also limitations in the data. First, compared to some previously published scRNA-seq studies of other species, the total numbers of cells in the current study are relatively low. Second, similar to other previously published studies on testicular cells, early developmental stages of germ cells and somatic populations are under-represented in current study, which may limit the data usage for these populations.

The raw data in.fastq format^22^ can be used as input for the Cellranger or similar tools. The gene-barcode matrices^23^ can be processed by the Seurat R package. As the RNA content of individual germ cells in the rodent testis varies greatly, it may be necessary to do an appropriate normalization, such as one based on the UMI count per cell, when comparing the current dataset to bulk RNA-Seq results from sorted cells or from whole testis.

## Supplementary information

Supplementary Materials

## Data Availability

No special code was used for analysis of the current dataset. All of the analyses were done with the following open access programs: FastQC version 0.11.9. (https://github.com/s-andrews/FastQC). Trimmomatic-0.39 program. (http://www.usadellab.org/cms/?page=trimmomatic). Cell Ranger version 2.1.1 (https://github.com/10XGenomics/cellranger). Seurat (https://satijalab.org/seurat). The Loupe Cell Browser v5.0.0 (https://www.10xgenomics.com/products/loupe-browser)

## References

[1] França LR (2016). The Sertoli cell: one hundred fifty years of beauty and plasticity. Andrology.

[2] Holdcraft RW, Braun RE (2004). Androgen receptor function is required in Sertoli cells for the terminal differentiation of haploid spermatids. Development.

[3] Zhou R (2019). The roles and mechanisms of Leydig cells and myoid cells in regulating spermatogenesis. Cell Mol. Life Sci..

[4] Jarow JP, Zirkin BR (2005). The androgen microenvironment of the human testis and hormonal control of spermatogenesis. Ann. NY Acad. Sci..

[5] Wang RS, Yeh S, Tzeng CR, Chang C (2009). Androgen receptor roles in spermatogenesis and fertility: lessons from testicular cell-specific androgen receptor knockout mice. Endocr. Rev..

[6] Lukassen S, Bosch E, Ekici AB, Winterpacht A (2018). Characterization of germ cell differentiation in the male mouse through single-cell RNA sequencing. Sci. Rep..

[7] Green CD (2018). A comprehensive roadmap of murine spermatogenesis defined by single-cell RNA-seq. Dev. Cell.

[8] Tan K, Song HW, Wilkinson MF (2020). Single-cell RNAseq analysis of testicular germ and somatic cell development during the perinatal period. Development.

[9] Shami AN (2020). Single-cell RNA sequencing of human, macaque, and mouse testes uncovers conserved and divergent features of mammalian spermatogenesis. Dev. Cell.

[10] Hermann BP (2018). The mammalian spermatogenesis single-cell transcriptome, from spermatogonial stem cells to spermatids. Cell Rep..

[11] Li L (2017). Single-cell RNA-seq analysis maps development of human germline cells and gonadal niche interactions. Cell Stem Cell..

[12] Sohni A (2019). The neonatal and adult human testis defined at the single-cell level. Cell Rep..

[13] Wang YM (1989). The androgen-binding protein gene is expressed in CD1 mouse testis. Mol. Cell. Endocrinol..

[14] Oduwole OO (2018). Constitutively active follicle-stimulating hormone receptor enables androgen-independent spermatogenesis. J. Clin. Invest..

[15] Huhtaniemi I (2018). Mechanisms in endocrinology: Hormonal regulation of spermatogenesis: mutant mice challenging old paradigms. Eur. J. Endocrinol..

[16] Coviello AD (2004). Intratesticular testosterone concentrations comparable with serum levels are not sufficient to maintain normal sperm production in men receiving a hormonal contraceptive regimen. J. Androl..

[17] Zirkin BR, Santulli R, Awoniyi CA, Ewing LL (1989). Maintenance of advanced spermatogenic cells in the adult rat testis: quantitative relationship to testosterone concentration within the testis. Endocrinology.

[18] Chen H, Stanley E, Jin S, Zirkin BR (2010). Stem Leydig cells: from fetal to aged animals. Birth Defects Res. C. Embryo Today.

[19] Klinefelter GR, Hall PF, Ewing LL (1987). Effect of luteinizing hormone deprivation *in situ* on steroidogenesis of rat Leydig cells purified by a multistep procedure. Biol. Reprod..

[20] Blondel VD (2008). Local leaders in random networks. Phys. Rev. E Stat. Nonlin. Soft Matter Phys..

[21] (2021). Genome Sequence Archive PRJCA006139.

[22] Chen H (2021). Ngdc omix.

[23] Chen H, Guan X (2022). figshare.

[24] Soumillon M (2013). Cellular source and mechanisms of high transcriptome complexity in the mammalian testis. Cell reports.

[25] Johnston DS (2008). Stage-specific gene expression is a fundamental characteristic of rat spermatogenic cells and Sertoli cells. Proc. Natl. Acad. Sci. USA.

[26] Phillips BT, Gassei K, Orwig KE (2010). Spermatogonial stem cell regulation and spermatogenesis. Phil. Trans. R. Soc. B..

[27] Ballow DJ, Xin Y, Choi Y, Pangas SA, Rajkovic A (2006). Sohlh2 is a germ cell-specific bHLH transcription factor. Gene Expr. Patterns.

[28] Prokai D (2020). Spermatogonial gene networks selectively couple to glutathione and pentose phosphate metabolism but not cysteine biosynthesis. iScience.

[29] Beverdam A (2009). Sox9-dependent expression of Gstm6 in Sertoli cells during testis development in mice. Reproduction.

[30] Ahmed M, Al-Daghri N, Alokail MS, Hussain T (2013). Potential changes in rat spermatogenesis and sperm parameters after inhalation of Boswellia papyrifera and Boswellia carterii incense. Int. J. Environ. Res. Public Health.

[31] El Shennawy A, Gates RJ, Russell LD (1998). Hormonal regulation of spermatogenesis in the hypophysectomized rat: cell viability after hormonal replacement in adults after intermediate periods of hypophysectomy. J. Androl..

