# Supplementary material for: Single-cell RNA sequencing of adult rat testes after Leydig cell elimination and restoration
Source: Sci Data. 2022 Mar 25;9:106. doi: 10.1038/s41597-022-01225-5 (PMC8956705; doi:10.1038/s41597-022-01225-5)
Supplement: Supplementary file 1 — Supplementary Materials [file 41597_2022_1225_MOESM1_ESM.pdf]

Supplemental Materials for Guan et al.,
Data Descriptor: Single-cell RNA sequencing of adult rat testes after Leydig cell elimination and restoration

There are 9 supplemental figures and 2 supplemental tables included

Supplemental Fig S1. Median Genes per Cell

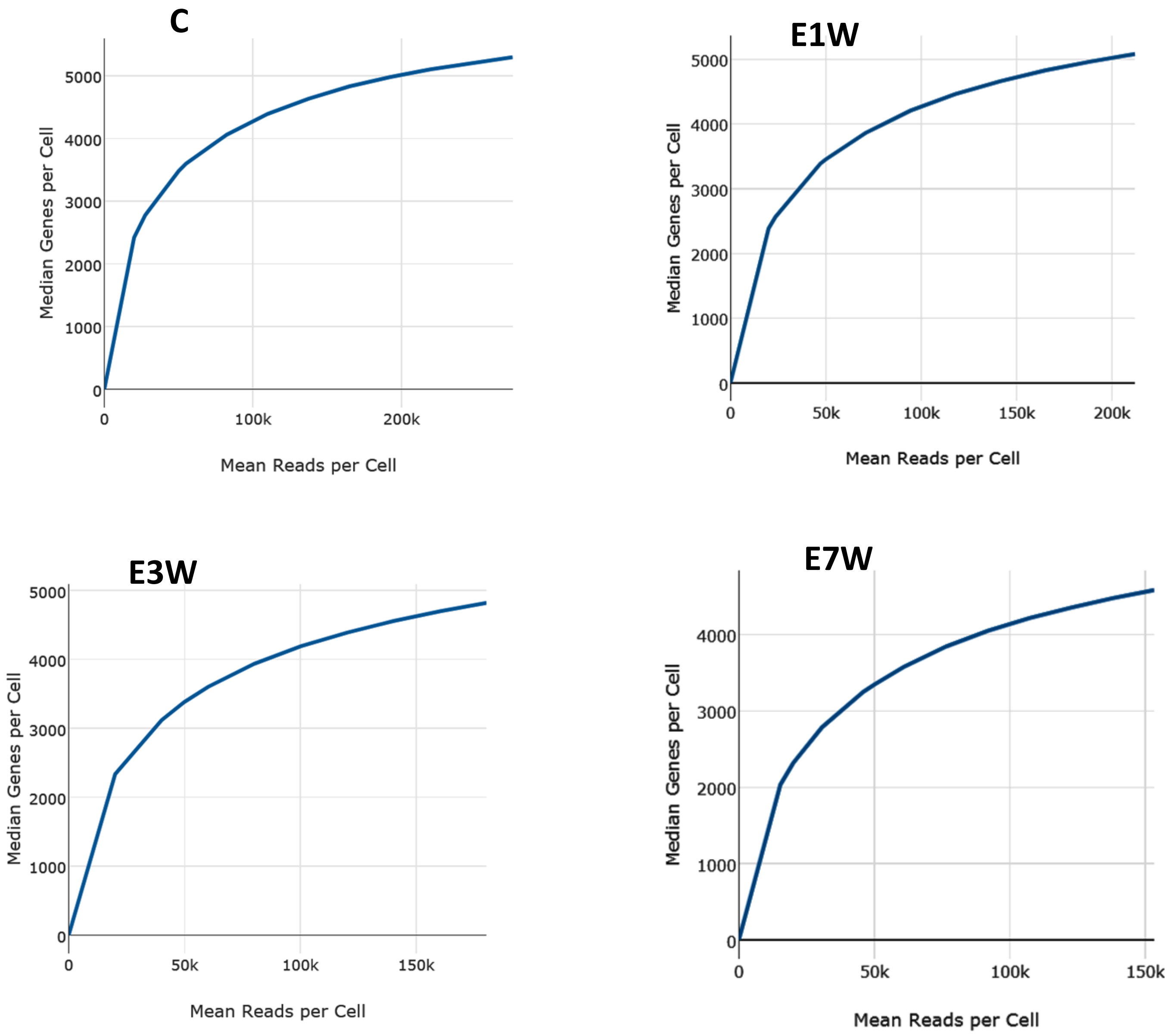

Supplemental Fig S2. Data Quality Control of Sample C

Estimated Number of Cells

2,157

Mean Reads per Cell	Median Genes per Cell
274,804	5,297

Sequencing

Number of Reads	592,752,404
Valid Barcodes	97.0%
Sequencing Saturation	50.3%
Q30 Bases in Barcode	94.6%
Q30 Bases in RNA Read	89.8%
Q30 Bases in UMI	94.4%

Mapping

Reads Mapped to Genome	93.7%
Reads Mapped Confidently to Genome	87.3%
Reads Mapped Confidently to Intergenic Regions	15.4%
Reads Mapped Confidently to Intronic Regions	8.1%
Reads Mapped Confidently to Exonic Regions	63.8%
Reads Mapped Confidently to Transcriptome	57.3%
Reads Mapped Antisense to Gene	0.8%

Cells

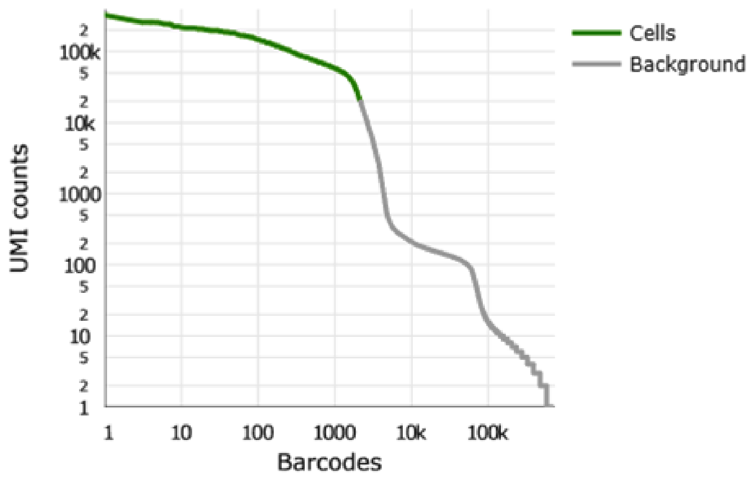

Estimated Number of Cells	2,157
Fraction Reads in Cells	84.7%
Mean Reads per Cell	274,804
Median Genes per Cell	5,297
Total Genes Detected	19,131
Median UMI Counts per Cell	55,914

Sample

Name	C
Description	
Transcriptome	Rattus_norvegicus
Chemistry	Single Cell 3' v2
Cell Ranger Version	2.1.1

Supplemental Fig S3. Data Quality Control of Sample E1W

Estimated Number of Cells

2,294

Mean Reads per Cell	Median Genes per Cell
235,716	5,183

Sequencing

Number of Reads	540,734,237
Valid Barcodes	96.7%
Sequencing Saturation	45.0%
Q30 Bases in Barcode	95.1%
Q30 Bases in RNA Read	90.3%
Q30 Bases in UMI	94.9%

Mapping

Reads Mapped to Genome	93.6%
Reads Mapped Confidently to Genome	87.3%
Reads Mapped Confidently to Intergenic Regions	15.6%
Reads Mapped Confidently to Intronic Regions	7.9%
Reads Mapped Confidently to Exonic Regions	63.8%
Reads Mapped Confidently to Transcriptome	57.1%
Reads Mapped Antisense to Gene	0.9%

Cells

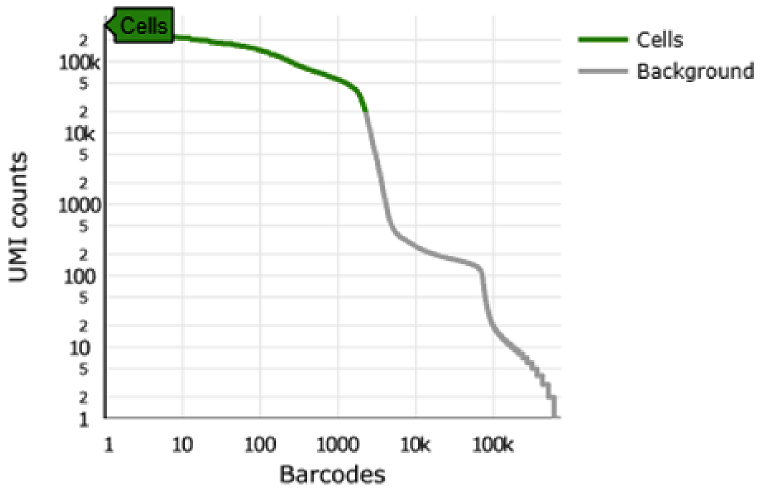

Estimated Number of Cells	2,294
Fraction Reads in Cells	84.6%
Mean Reads per Cell	235,716
Median Genes per Cell	5,183
Total Genes Detected	19,153
Median UMI Counts per Cell	52,393

Sample

Name	E1W
Description	
Transcriptome	Rattus_norvegicus
Chemistry	Single Cell 3' v2
Cell Ranger Version	2.1.1

Supplemental Fig S4. Data Quality Control of Sample E3W

Estimated Number of Cells

2,839

Mean Reads per Cell	Median Genes per Cell
200,468	4,925

Sequencing

Number of Reads	569,130,075
Valid Barcodes	96.8%
Sequencing Saturation	42.9%
Q30 Bases in Barcode	94.6%
Q30 Bases in RNA Read	89.5%
Q30 Bases in UMI	94.4%

Mapping

Reads Mapped to Genome	94.0%
Reads Mapped Confidently to Genome	87.4%
Reads Mapped Confidently to Intergenic Regions	15.1%
Reads Mapped Confidently to Intronic Regions	8.1%
Reads Mapped Confidently to Exonic Regions	64.3%
Reads Mapped Confidently to Transcriptome	57.5%
Reads Mapped Antisense to Gene	1.0%

Cells

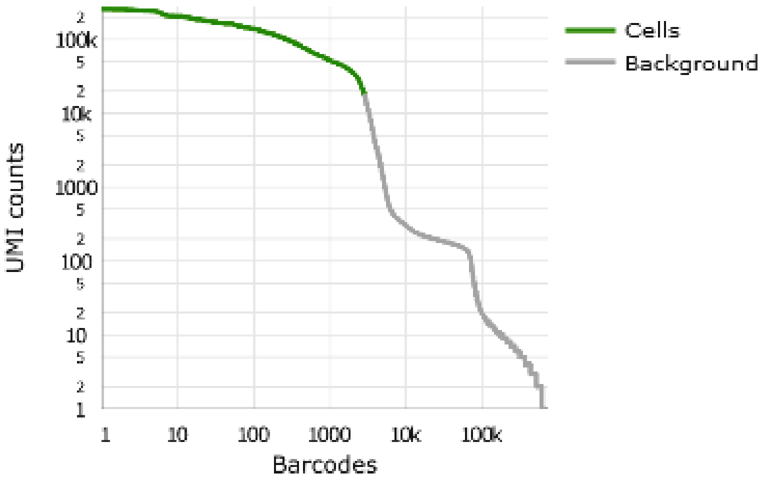

Estimated Number of Cells	2,839
Fraction Reads in Cells	84.1%
Mean Reads per Cell	200,468
Median Genes per Cell	4,925
Total Genes Detected	19,284
Median UMI Counts per Cell	44,653

Sample

Name	E3W
Description	
Transcriptome	Rattus_norvegicus
Chemistry	Single Cell 3' v2
Cell Ranger Version	2.1.1

Supplemental Fig S5. Data Quality Control of Sample E7W

Estimated Number of Cells

3,693

Mean Reads per Cell	Median Genes per Cell
153,305	4,585

Sequencing

Number of Reads	566,156,197
Valid Barcodes	96.7%
Sequencing Saturation	39.2%
Q30 Bases in Barcode	94.5%
Q30 Bases in RNA Read	89.6%
Q30 Bases in UMI	94.3%

Mapping

Reads Mapped to Genome	94.0%
Reads Mapped Confidently to Genome	87.8%
Reads Mapped Confidently to Intergenic Regions	15.3%
Reads Mapped Confidently to Intronic Regions	7.8%
Reads Mapped Confidently to Exonic Regions	64.7%
Reads Mapped Confidently to Transcriptome	57.5%
Reads Mapped Antisense to Gene	1.1%

Cells

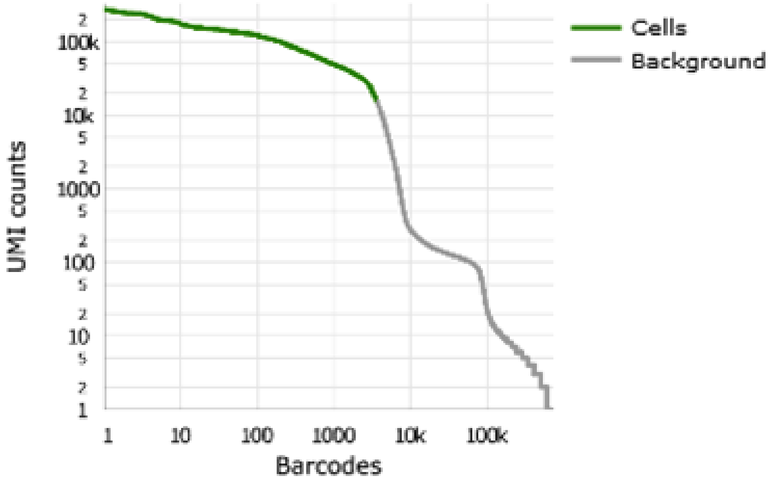

Estimated Number of Cells	3,693
Fraction Reads in Cells	84.1%
Mean Reads per Cell	153,305
Median Genes per Cell	4,585
Total Genes Detected	19,324
Median UMI Counts per Cell	36,562

Sample

Name	E7W
Description	
Transcriptome	Rattus_norvegicus
Chemistry	Single Cell 3' v2
Cell Ranger Version	2.1.1

Supplemental Fig S6. Dot- and Violin-Plots Showing the Distributions of nCount_RNA, nFeature_RNA, Percent.mito and Percent.HB

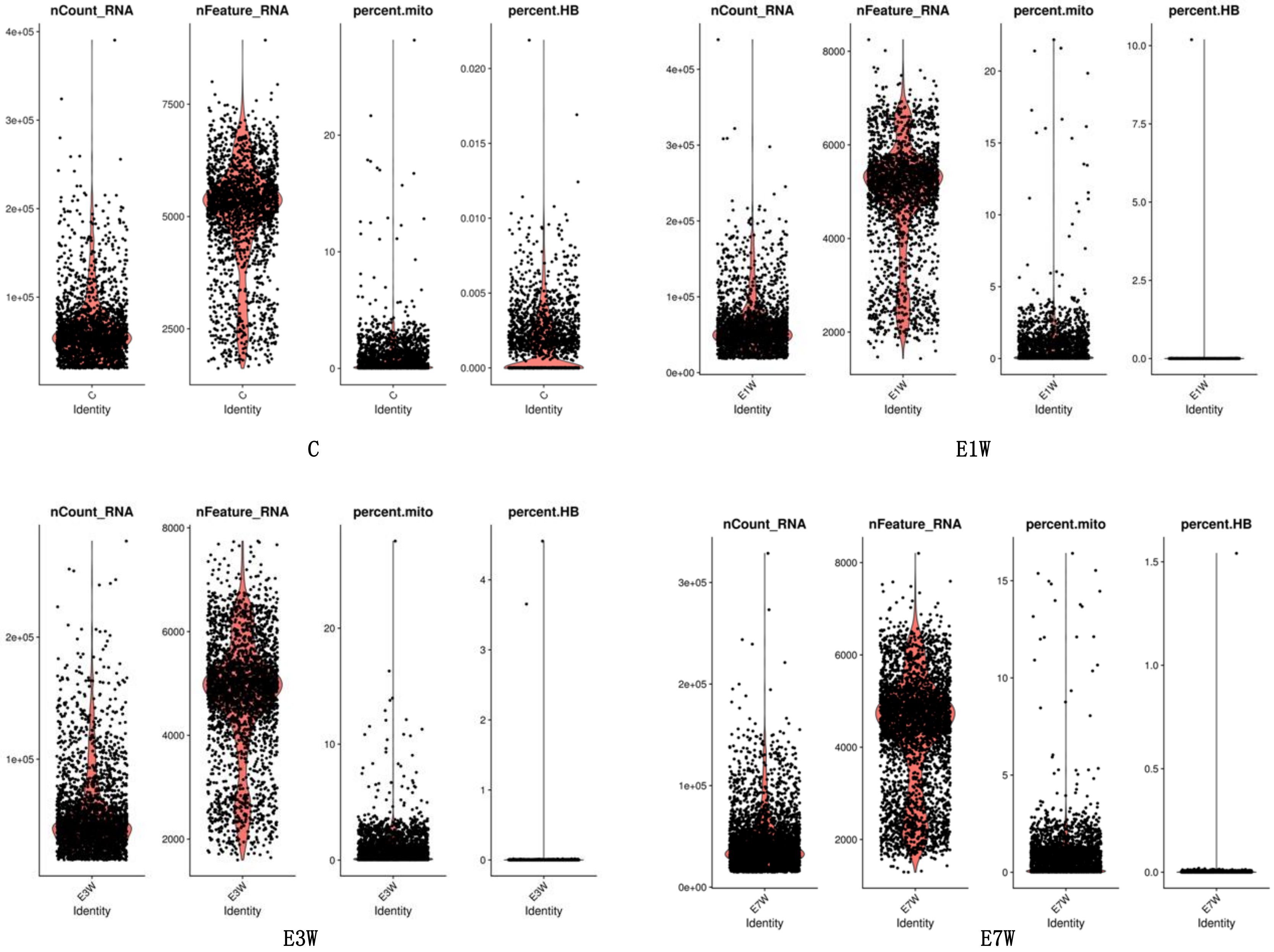

Supplemental Fig S7. Violin Plots of Genes Specifically Expressed by Different Testicular Cell Types

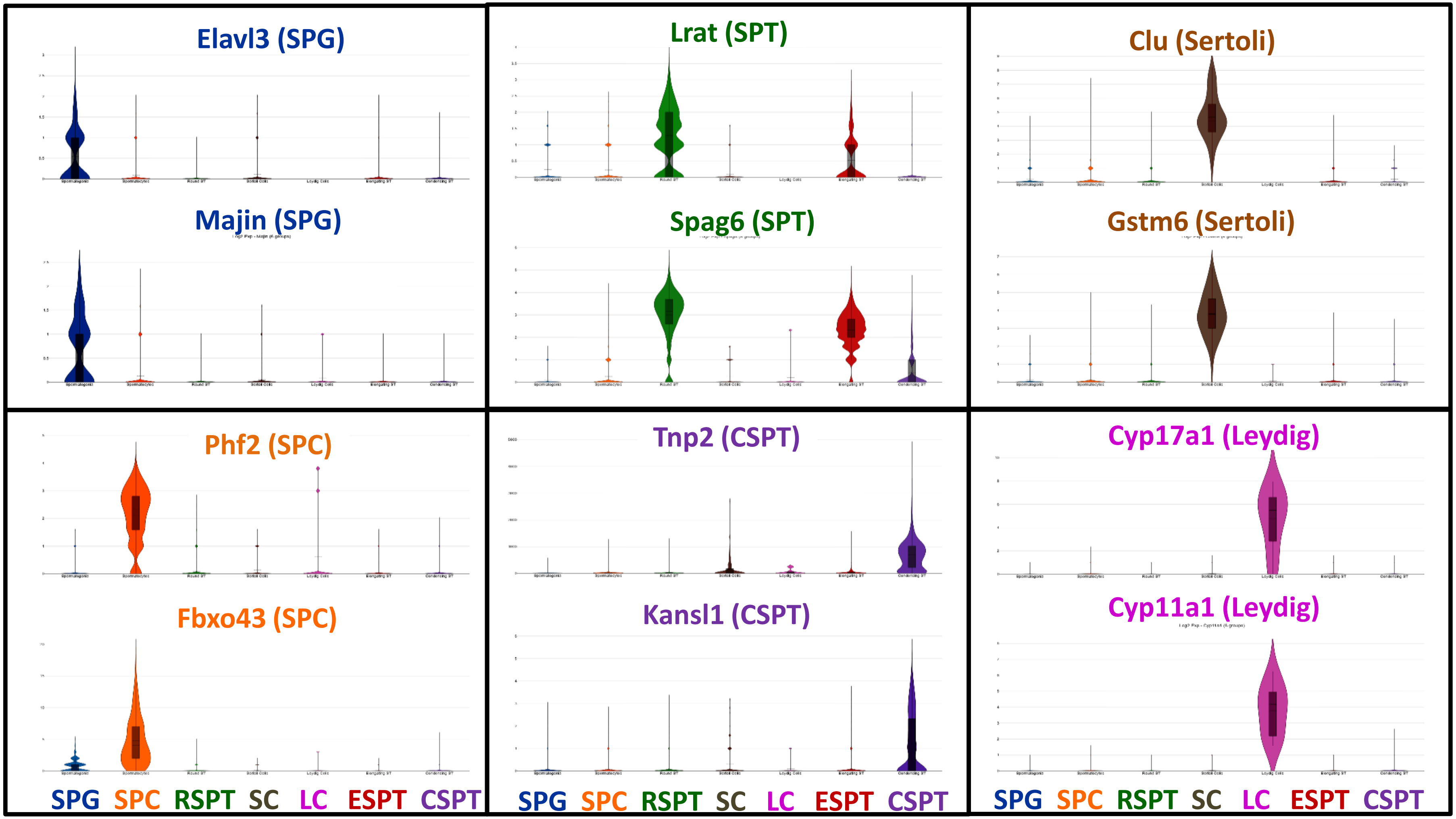

Supplemental Fig S7. Violin plots of genes specifically expressed by different testicular cell types. SPG: Spermatogonia; SPC: Spermatocytes; RSPT: Round spermatids; SC: Sertoli cells; LC: Leydig cells; ESPT: Elongating spermatids; CSPT: Condensing spermatids.

Supplemental Fig S8. Violin Plots of Genes Specifically Expressed by Different Germ Cell Types

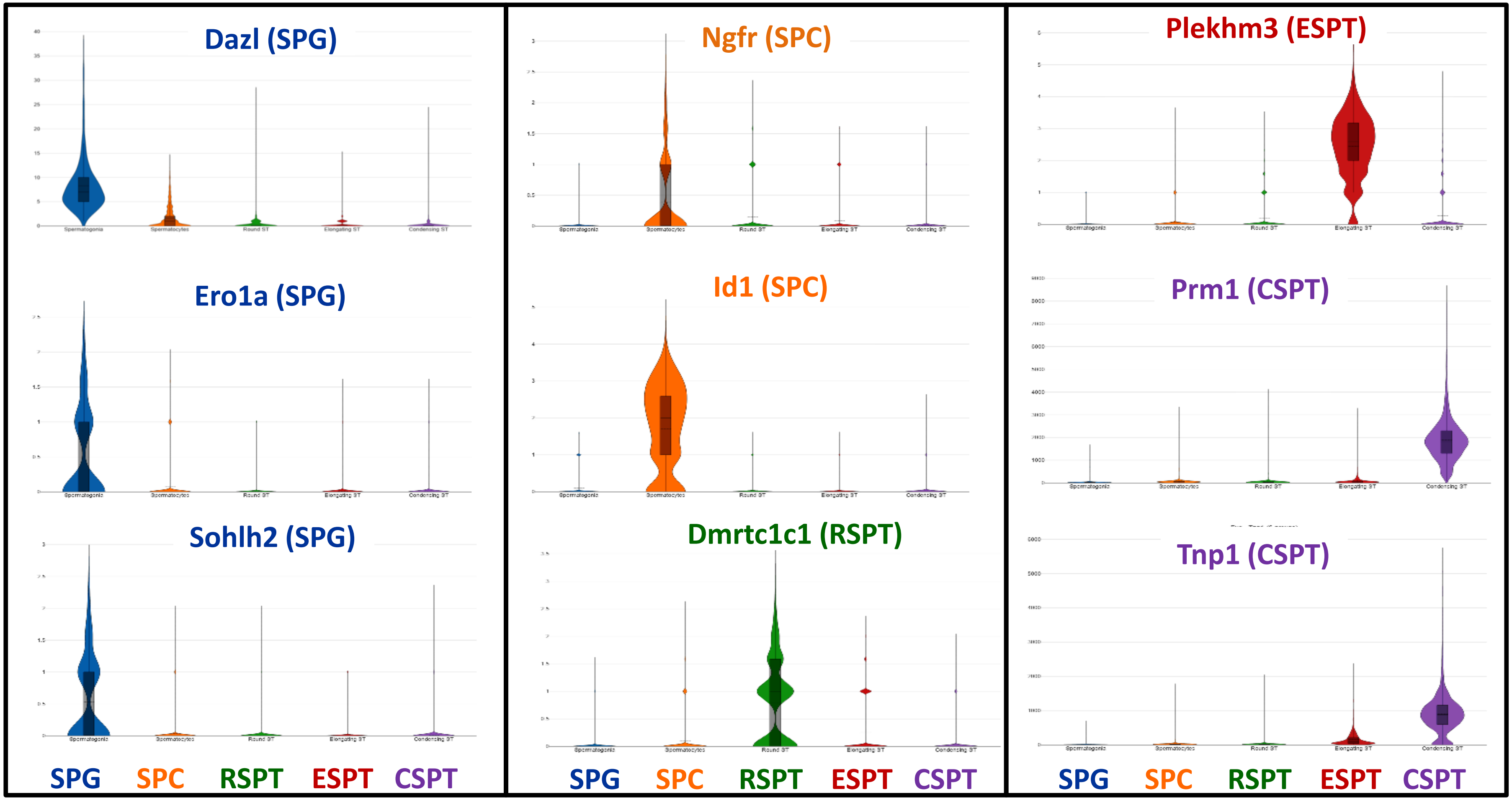

Supplemental Fig S8. Violin plots of genes specifically expressed by different developmental germ cells. SPG: Spermatogonia; SPC: Spermatocytes; RSPT: Round spermatids; ESPT: Elongating spermatids; CSPT: Condensing spermatids.

Supplemental Fig S9. Expression of Gfra1 by Germ Cells of Different Developmental Stages

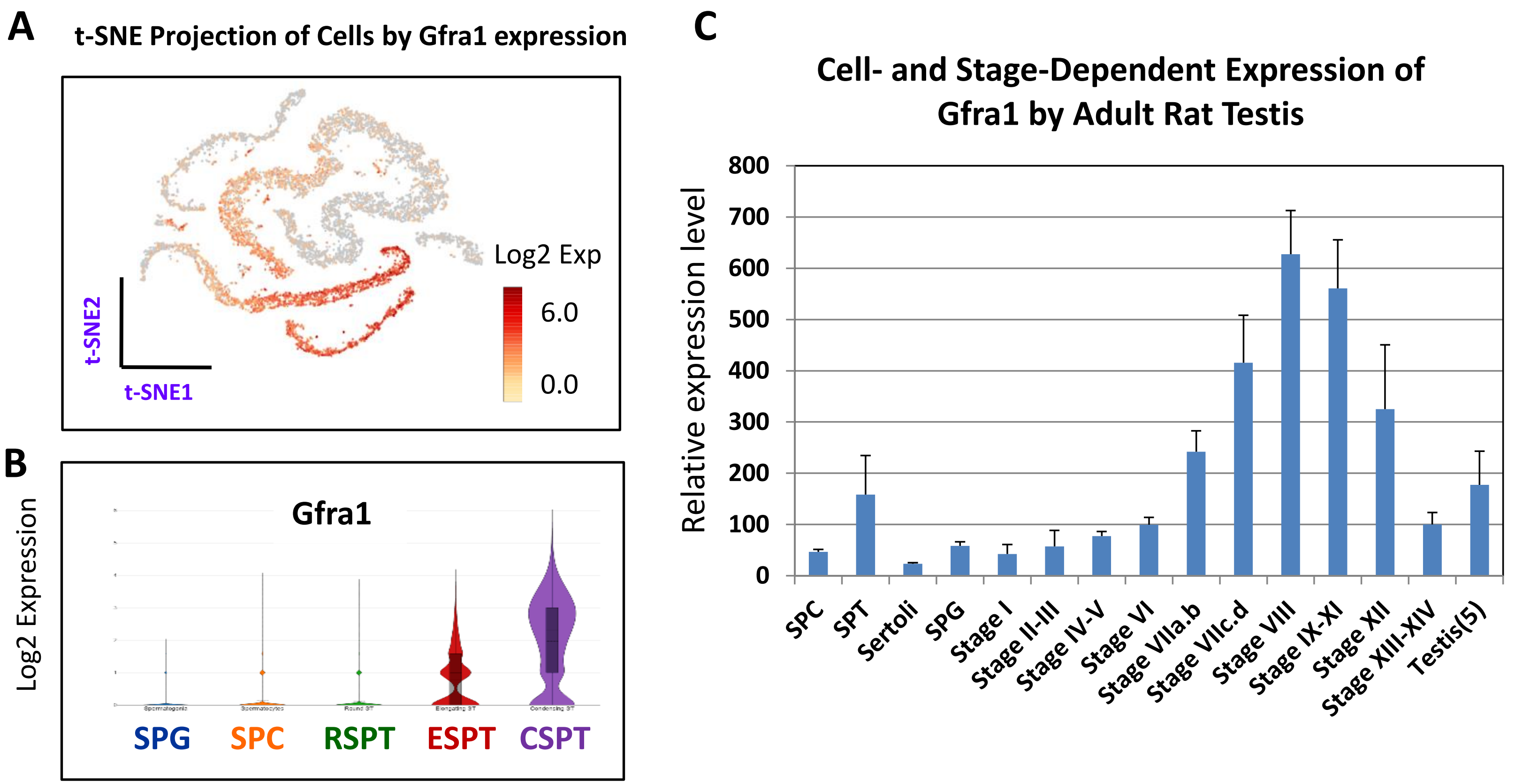

Supplemental Fig S9. Expression of Gfra1 by developing germ cells and seminiferous tubules of spermatogenic stages. A. t-SNE plot of Gfra1 expression by testicular cells; B. Violin plot of Gfra1 expression by developing germ cells. C. Expression of Gfra1 by testicular cells and seminiferous tubules of adult rat. The graph C is redrawn from a microarray dataset of a previously publication (Johnston et al., 2008)[22] with permission. SPG: Spermatogonia; SPC: Spermatocytes; SPT: Spermatids; RSPT: Round spermatids; ESPT: Elongating spermatids; CSPT: Condensing spermatids.

[22]Johnston et al., 2008. Proc Natl Acad Sci U S A. 105(24):8315-20. doi: 10.1073/pnas.0709854105.

Supplemental Table S1. Number of Genes Shared Among the Top 200 Differentially Expressed Genes (DEGs) for the Major Testicular Cells of 3 scRNA-Seq Studies.

DEGs shared between Green and current studies

A

mice\rat	SPG	SPC	RST	EST	Sertoli	Leydig
SPG	**17**	4	7	1	3	2
SPC	2	**13**	5	0	0	0
RST	0	0	**27**	1	0	0
EST	0	0	0	**33**	0	0
Sertoli	1	3	3	2	**49**	2
Leydig	6	0	1	3	12	**51**

Mice: Green study[7]
Rat: Current study

DEGs shared between Harmann and current studies

B

mice\rat	SPG	SPC	RST	EST	Sertoli	Leydig
SPG	**17**	4	2	1	5	6
SPC	8	**1**	6	2	9	14
RST	1	0	**0**	**24**	0	0

Mice: Harmann study[10]
Rat: Current study

DEGs shared between Harmann and current studies

C

man\rat	SPG	SPC	RST	EST	Sertoli	Leydig
SPG	**4**	4	4	2	5	4
SPC	3	**15**	2	1	1	1
RST	1	2	**0**	**17**	2	0

Man: Harmann study[10]
Rat: Current study

[7]Green, C. D. et al. Dev. Cell 46, 651-667.e10, https://doi.org/10.1016/j.devcel.2018.07.025 (2018).
[10]Hermann, B. P. et al. Cell Rep. 25,1650-1667.e8, https://doi.org/10.1016/j.celrep.2018.10.026 (2018).

Supplemental Table S2. Number of Genes Shared Among the Top 200 Differentially Expressed Genes (DEGs) for the Major Testicular Cells of 2 scRNA-Seq Studies.

A

DEGs shared between mice germ cells of Green and Harmann studies

mice1 \ mice2	SPG	SPC	RSPT	ESPT	Sertoli	Leydig
SPG	**51**	**0**	0	1	7	15
SPC	53	**0**	0	2	9	21
SPT	0	0	**7**	**55**	0	1

Mice1: Harmann study[10]
Mice2: Green study[7]

B

DEGs shared between mice and human germ cells of Harmann study

mice \ Man	SPG	SPC	SPT
SPG	**18**	5	4
SPC	21	**0**	3
SPT	2	1	**24**

Mice: Harmann study[10]
Man: Harmann study[10]

[7]Green, C. D. et al. Dev. Cell 46, 651-667.e10, https://doi.org/10.1016/j.devcel.2018.07.025 (2018).
[10]Hermann, B. P. et al. Cell Rep. 25,1650-1667.e8, https://doi.org/10.1016/j.celrep.2018.10.026 (2018).
